# Optimal modes of mind-body exercise for treating chronic non-specific low back pain: Systematic review and network meta-analysis

**DOI:** 10.3389/fnins.2022.1046518

**Published:** 2022-11-17

**Authors:** Jian Shi, Zheng-Yu Hu, Yu-Rong Wen, Ya-Fei Wang, Yang-Yang Lin, Hao-Zhi Zhao, You-Tian Lin, Yu-Ling Wang

**Affiliations:** ^1^College of Kinesiology, Shenyang Sport University, Shenyang, China; ^2^Rehabilitation Medicine Center, The Sixth Affiliated Hospital, Sun Yat-sen University, Guangzhou, China; ^3^Department of Sport Rehabilitation, Shanghai University of Sport, Shanghai, China; ^4^Postgraduate Research Institute, Guangzhou Sport University, Guangzhou, China

**Keywords:** tai chi, yoga, qigong, Pilates, mind-body exercise, chronic low back pain, network meta-analysis

## Abstract

**Background:**

There were limited studies that directly compare the outcomes of various mind-body exercise (MBE) therapies on chronic non-specific low back pain (CNLBP).

**Objectives:**

To compare the efficacy of the four most popular MBE modes [Pilates, Yoga, Tai Chi (TC), and Qigong] in clinically CNLBP patients, we conducted a systematic review and network meta-analysis (NMA).

**Methods:**

We searched databases for eligible randomized controlled trials (RCTs) (from origin to July 2022). RCTs were eligible if they included adults with CNLBP, and implemented one or more MBE intervention arms using Pilates, yoga, TC, and qigong. In addition, pain intensity and physical function were evaluated using validated questionnaires.

**Results:**

NMA was carried out on 36 eligible RCTs involving 3,050 participants. The effect of exercise therapy on pain was in the following rankings: Pilates [Surface under cumulative ranking (SUCRA) = 86.6%], TC (SUCRA = 77.2%), yoga (SUCRA = 67.6%), and qigong (SUCRA = 64.6%). The effect of exercise therapy on function: Pilates (SUCRA = 98.4%), qigong (SUCRA = 61.6%,), TC (SUCRA = 59.5%) and yoga (SUCRA = 59.0%).

**Conclusion:**

Our NMA shows that Pilates might be the best MBE therapy for CNLBP in pain intensity and physical function. TC is second only to Pilates in improving pain in patients with CNLBP and has the value of promotion. In the future, we need more high-quality, long-term follow-up RCTs to confirm our findings.

**Systematic review registration:**

https://www.crd.york.ac.uk/PROSPERO/display_record.php?RecordID=306905, identifier: CRD42022306905.

## Introduction

Low back pain (LBP), which occurs below the costal border and above the buttock folds, is one of the most prevalent public health issues worldwide (Van Tulder et al., 2006). Non-specific LBP (NLBP) refers to LBP for which no clear cause has been found and accounts for approximately 80–90% of all cases of LBP (Casazza, 2012), and a significant proportion of patients (10–20%) develop chronic NLBP (CNLBP) lasting at least 12 weeks (Maher et al., 2017). LBP is a major risk factor for physical disability globally, thus affecting nearly 20–25% of the global population over the age of 65 (Vadalà et al., 2020). In the United States, the total annual fiscal effect of low back and neck pain is the third-highest proportion of health care expenditures (Dieleman et al., 2016) and it affects approximately 13.1% of adults from 20 to 69 years old (Shmagel et al., 2016). However, satisfice with treatment is low for CNLBP patients (Patrick et al., 2014). In addition, CNLBP patients usually have a high recurrence rate (Taylor et al., 2014) and were associated with an increased risk of comorbidities such as depression and anxiety (Taylor et al., 2014). Conventional drug therapy appears to provide a short-term benefit to the symptoms of patients with CNLBP; however, recent studies have questioned the effectiveness and safety of these interventions (Deyo et al., 2015b; Al-Qurain et al., 2020; Cashin et al., 2021). Meanwhile, long-term use of analgesics is associated with psychopathy-like depression (Maher et al., 2017) and may decrease bone mass and induce sexual dysfunction (Bishop and Wing, 2003). Pharmacotherapy is insufficient to resolve chronic pain symptoms and improve physical function for this population. Therefore, recently, various clinical guidelines have recommended that the treatment of CNLBP should focus on non-pharmacological interventions (Bernstein et al., 2017; Qaseem et al., 2017; Stochkendahl et al., 2018).

Over the past decades, the advantages of exercise therapy have been discovered in the literature (Miyamoto et al., 2019; Hayden et al., 2020, 2021a; Owen et al., 2020), and it has been used as a first-line option to treat CNLBP (Chiarotto and Koes, 2022). Mind-body exercise (MBE), is a mild to moderate intensity physical activity, such as tai chi (TC) (Qin et al., 2019), yoga (Zhu et al., 2020), qigong (Li et al., 2019) (e.g., Baduanjin and Wuqinxi), and Pilates (Miyamoto et al., 2013), has attracted researchers' wide attention (Zou et al., 2019; Wen et al., 2022). MBE underlines mind-body integration and has the advantages of both mind-body therapy and exercise therapy. It involves various slow body movements synchronized with musculoskeletal relaxation, breathing control, and a meditative state of mind (Bower and Irwin, 2016; Zou et al., 2018). In recent years, it has been successfully used worldwide for the treatment of CNLBP (Teut et al., 2016; Cruz-Díaz et al., 2018; Liu et al., 2019; Yao et al., 2020) and is recommended as a complementary and alternative medicine therapeutic intervention based on the guidelines of the American College of Physicians (Qaseem et al., 2017). Moreover, some meta-analyses indicated that MBE is beneficial for pain intensity and back-specific disability of patients with CNLBP (Li et al., 2019; Qin et al., 2019; Zou et al., 2019; Anheyer et al., 2022). Evidence for these results was also supplied in our previous study (Wen et al., 2022).

Although there is some evidence that MBE intervention is effective in treating the symptoms of patients with CNLBP, there are varying modes. The low efficacy of MBE intervention not only delays the CNLBP patients' condition but also increases unnecessary medical costs. It has become a critical task to further rank the efficacy of different forms of MBE to obtain more comprehensive evidence in terms of MBE for improving symptoms of CNLBP. However, there has been little effort to compare the curative effect of different MBE modes to obtain a deeper awareness. Most randomized controlled trials (RCTs) compare MBE interventions with no treatment or usual care groups, and direct comparisons between different MBE modes were very few. Based on our search results, only one RCT direct compared yoga with qigong in the treatment of patients with CNLBP (Teut et al., 2016). It is because, a head-to-head comparative study would be very expensive, and it would be impractical to use an RCT to examine the relative effects of all MBE modes. Meta-analyses provide a summary estimate of treatment effects by combining data from various studies. However, an important drawback is that standard meta-analyses can only compare two interventions at a time. Meanwhile, network meta-analysis (NMA) can indirectly compare multiple treatments by a common comparator to synthesize evidence across a network of RCTs. Therefore, researchers will be able to rank the effectiveness of multiple MBE modes by the use of NMA.

To date, limited reviews and NMA were done on exercise for patients with CNLBP (Owen et al., 2020; Hayden et al., 2021b; Fernández-Rodríguez et al., 2022). Owen et al. (2020) accomplished a sequential analysis and NMA to evaluate whether or not there was ample evidence to support the application of physical exercise for CNLBP patients and whether one exercise mode was better than another. But TC was included in “Other exercise” intervention group and Qigong-related studies were not included in their NMA. Similar classification appears in the studies of Hayden et al. (2021b) and Fernández-Rodríguez et al. (2022). We cannot find out which MBE mode is the most optimal for improving pain intensity and physical function of patients with CNLBP through current studies. Moreover, most NMA does not include Chinese RCTs because of language barriers and limited retrieval resources. Therefore, it is necessary to identify and assess the best MBE modes for CNLBP treatment by a new systematic review and NMA.

This review aimed to conduct a systematic review and NMA of current evidence from RCTs to compare the therapeutic effects of four common MBE modes (TC, yoga, qigong, and Pilates) in improving pain intensity and physical function for adults with CNLBP. The results of this review may help clinicians choose the ideal MBE modes for the treatment of CNLBP and enrich the theoretical basis for MBE selection. Meanwhile, for patients, the results of this study are assumed to provide evidence-based advice for treatment planning for them and to use optimal MBE intervention as the ideal form of self-care to relieve their symptoms and improve physical function.

## Materials and methods

### Protocol and registration

In the International Prospective Register of Systematic Reviews, the protocol was prospectively recorded (CRD42022306905) and was conducted by Preferred Reporting Items for Systematic Reviews and Meta-Analysis for Network Meta-Analysis (PRISMA-NMA) (Hutton et al., 2015).

### Literature search

This search strategy was designed using systematic reviews (Zou et al., 2019; Owen et al., 2020; Wen et al., 2022) that have already published and the Cochrane Back and Neck Group (Furlan et al., 2015). It was based on the following seven databases, including PubMed, Embase, Web of Science, Cochrane Library, China National Knowledge Infrastructure (CNKI), Wanfang Database, and Chinese Scientific Journals Full-Text Database (VIP). Publication dates ranged from the first date available to July 2022 in all languages. Moreover, the following keywords are searched: “Mind-body exercise,” “Tai chi”, “Yoga”, “Pilates”, “Qigong”, and “Chronic low back pain”. The complete searching strategies of all databases are submitted in Supplementary material 1.

### Eligibility criteria

Participants, Intervention, Comparison, Outcomes, and Study (PICOS) design was employed as a framework to enact eligibility criteria (Hutton et al., 2015).

#### Inclusion criteria

(1) adults (≥18 years) that were diagnosed with CNLBP at baseline based on the National Institutes of Health (NIH) definition (Deyo et al., 2015a).
(2) to assess the therapeutic impact of one or even more MBE arms, an RCT protocol was adopted.
(3) to avoid the influence of different positive background treatments between the MBE group and the control group on the final NMA results, MBE group only received TC, yoga, qigong, or Pilates intervention with no additional treatments (e.g., electrotherapy, manipulation). For the NMA, we need to include a common comparator across different MBE modes. The common comparator refers to the comparator which has been used by at least two studies for two different exercises (Li et al., 2011; Goh et al., 2016). The control group included no treatment control, usual care control, and conventional therapeutic exercise control.
(4) at least one of the outcome measures of interest were included in studies: subjective pain intensity and subjective physical function level.

#### Exclusion criteria

(1) conference abstracts, researcher protocols, and books all published studies.
(2) study data could not be obtained or converted.
(3) recruited patients suffering from acute, subacute LBP or LBP with unclear duration (e.g., recurrent LBP without a clear duration).
(4) LBP due to pregnancy, infections, tumors, osteoporosis, fractures, structural malformations (such as scoliosis), inflammatory disorders, radiculopathy, or cauda equina syndrome are excluded.
(5) trials were excluded if pain intensity and disability were not considered as primary or secondary outcomes.

#### Data extraction

Here, two evaluators (JS and HZZ) independently extracted data from each chosen study using a data extraction form, and then reviewed and revised by the corresponding author, including publication information (e.g., author, year, and country of origin), study design (e.g., parallel or crossover trail, two- or multi-arm parallel trial), subject characteristics (e.g., age, gender, pain duration, and sample size), interventions considered (e.g., TC, yoga, qigong, and Pilates), and outcome measures (e.g., pain intensity and physical function). Considering the determinate baseline similarities of pain intensity and physical function measures in included RCTs, post-intervention mean and standard deviation (SD) were directly extracted as outcome data from the published data. However, when the necessary information could not be adequately extracted, we got in touch with the study's authors to request it. When standard errors (SEs), confidence intervals (Cls), or interquartile ranges (IQRs) were provided in place of Means and SDs, RevMan 5.3 calculator was used to convert these to Means and SDs. In addition, if data were expressed only as a graph (rather than numerical data within the text), the software Engauge Digitizer 10.8 was used to extract it. Meanwhile, when there were multiple post-intervention measurement points where data could be extracted such as post-intervention and follow-up, only data immediately following the end of the intervention stage was used.

### Risk of bias

The Cochrane Risk of Bias Tool (Sterne et al., 2019) was used to independently assess the methodological quality and the risk of bias of these studies by two authors (ZYH and YRW). In order to analyze potential selection bias, performance bias, detection bias, attrition bias, reporting bias, and other relevant biases, the Cochrane tool split the quality risk into three categories: low, high, and uncertain. Two assessors will reach a consensus through a discussion if there are any discrepancies regarding the risk of bias in these studies. However, when a consensus cannot be reached between two assessors, the corresponding author will give his opinion and adopts the consensus of the majority.

### Data synthesis and analysis

The NMA was performed using Stata v16.0 software (StataCorp, Texas, USA) based frequentist approach and in conformity with PRISMA-NMA guidelines (Shim et al., 2017). The crucial supposition underlying a network meta-analysis is that of network consistency, in other words, the therapeutic effects are equivalent on average, whether they are estimated by direct or indirect comparisons. Herein, the NMA's consistency was evaluated by fitting both the consistency and the inconsistency NMA and taking into account the outcomes of the Wald test for inconsistency. Moreover, the node-splitting technique was used to further evaluate inconsistency. Given the possibility of heterogeneity among studies, we choose the random effects model for the meta-analysis.

Standardized mean difference (SMD) was utilized as the summary measure to homogenize results from several scales and instruments into a single scale because all of the outcomes of interest were continuous or ordinal. When trails were inverted scaled (with higher values favoring outcomes instead of lower values), the mean in each group was multiplied by −1 as suggested by the Cochrane Handbook (Higgins et al., 2019) to guarantee all outcomes were illustrated with lower values, thereby suggesting improvements in pain intensity or physical function.

Herein, the interventions were ranked once their comparative effectiveness had been assessed to determine their superiority of the interventions. Surface under cumulative ranking (SUCRA) values, mean rank, and cumulative ranking plots for all outcomes were used to reflect the effects of different MBE to improve the values of pain intensity and physical function. The value of SUCRA ranges from 0 to 100% and a higher value indicates a greater possibility given that MBE mode is in the top rank or highly effective (Page et al., 2016). These data, which were averaged over the 10,000 replications, rank treatments according to their capacity to deliver the biggest treatment effects in each simulation. At least three studies on the same mode of MBE were required to rank the efficacy of interventions. Network funnel plots were generated and visually inspected using the symmetry criteria by us to examine for the presence of publication bias caused by small-scale studies that could contribute to publication bias in NMA. We also performed pairwise meta-analysis to compare the two interventions with pooled effect sizes. The value of the *I*^2^ statistic (*I*^2^ statistic whose values were 25, 50, and 75% indicated mild, moderate, and high heterogeneity) was used to assess the heterogeneity.

## Results

### Search results

**Figure 1:**
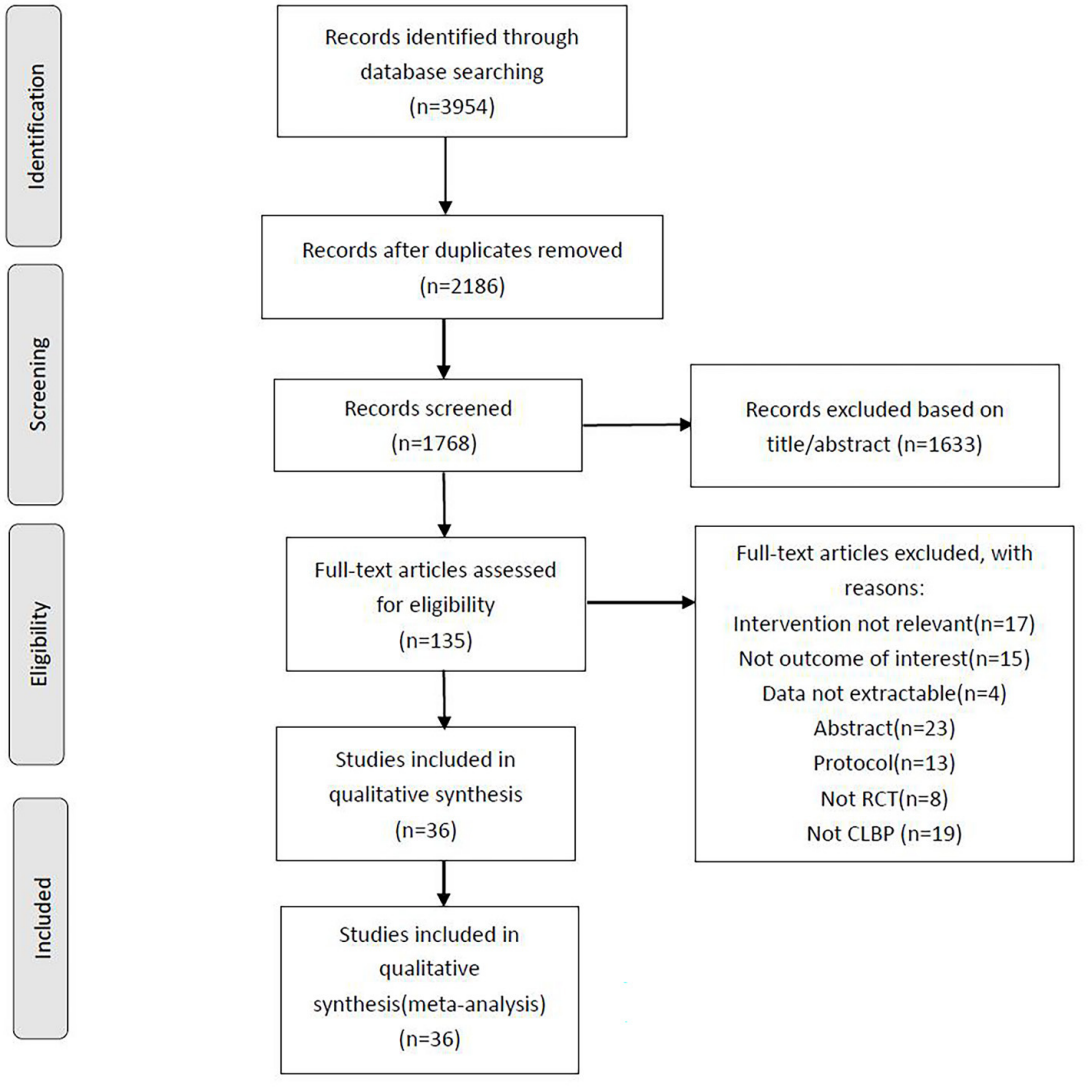
PRISMA flow diagram of the search process for studies examining the efficacy of mind-body exercise in patients with non-specific chronic low back pain.

A preliminary search of seven databases identified a total of 3,954 records. In the preliminary search results, there were 2,186 duplicate records excluded, and 1,633 records that did not match the review's inclusion criteria were eliminated based on the title and abstract. Then, through the evaluation of the full text of the remaining 135 studies, we found that 99 studies of them for several reasons, including intervention not relevant MBE (*n* = 17), not the outcome of interest (*n* = 15), data not extractable (*n* = 4), conference abstracts (*n* = 23), study protocol (*n* = 13), not RCT (*n* = 8), not CNLBP (*n* = 19). Ultimately, 36 studies were included in NMA. The systematic review process is shown in Figure 1.

### Study characteristics

The fundamental characteristics of all articles were summarized in Table 1. The considered studies were published from 2005 to 2021. Among the included studies, most of them were carried out in China (12/36) and the others were conducted in the USA (4/36), India (3/36), Germany (3/36), Spain (3/36), Korea (2/36), Australia (2/36), Brazil (1/36), Turkey (1/36), Iran (1/36), Thailand (1/36), Croatia (1/36), UK (1/36), and Italy (1/36). A total of 36 eligible RCTs with 3,050 subjects diagnosed with CNLBP were included in this NMA. Meanwhile, three studies (Kim et al., 2014; Kofotolis et al., 2016; Bai et al., 2020) included only females, and all others included both sexes. Furthermore, three studies (Lee et al., 2014; Patti et al., 2016; Mazloum et al., 2018) did not present information on gender distribution. Thus, researchers carried out various MBE treatments, which included yoga (Williams et al., 2005, 2009; Cox et al., 2010; Sherman et al., 2011; Tekur et al., 2012; Kim et al., 2014; Lee et al., 2014; Nambi et al., 2014; Teut et al., 2016; Saper et al., 2017; Kuvačić et al., 2018; Demirel et al., 2019; Neyaz et al., 2019; Bai et al., 2020; Michalsen et al., 2021) (studies: *n* = 15, subjects, *n* = 652), TC (Hall et al., 2011; He, 2013; Tong, 2017; Liu et al., 2018, 2019; Wang, 2020) (studies: *n* = 6, subjects, *n* = 183), qigong (Ding and Wang, 2014; Blödt et al., 2015; Ning et al., 2015; Teut et al., 2016; Wu, 2016; Wang, 2018; Phattharasupharerk et al., 2019; Chen, 2020; Yao et al., 2020) (studies: *n* = 9, subjects, *n* = 348), and Pilates (Wajswelner et al., 2012; Natour et al., 2015; Kofotolis et al., 2016; Patti et al., 2016; Valenza et al., 2017; Cruz-Díaz et al., 2018; Mazloum et al., 2018) (studies: *n* = 7, subjects, *n* = 205). There were three control comparators including no treatment, usual care, and conventional therapeutic exercises. The intervention duration of all MBE was between 1 and 24 weeks and sessions ranged from 4 to 96. A total of 32 studies used pain intensity as an outcome measure and the assessment scales were the Visual Analog Scale (VAS), Numeric Rating Scale (NRS), Defense and Veterans Pain Rating Scale (DVPRS), and Oswestry Disability Index (ODI)-pain. Meanwhile, 24 studies used physical function as an outcome measure and the assessment scales were ODI, Quebec Back Pain Disability Scale (QBPDS), and Roland Morris disability questionnaire (RMDQ).

**Table 1:** Principal characteristics of included studies.

**References**	**Country**	**Sample size (F/M)**	**Age, Mean (SD) years**	**Duration (weeks)**	**Follow-up weeks**	**Main pain/function outcome assessments**	**Experimental group intervention**	**Control group intervention**
Liu et al. (2019)	China	11/32	59.0(4.6)	12	–	VAS	Tai Chi (60 min/36 sessions)	CTE/NT
Hall et al. (2011)	Australia	119/41	43.9(13.2)	10	–	NRS	Tai Chi (40 min/18 sessions)	NT
Liu et al. (2018)	China	37/8	57.2(3.3)	12	–	ODI	Tai Chi (60 min/36 sessions)	NT
Wang (2020)	China	29/16	31.8(9.7)	6	12	NRS/RMDQ	Tai Chi (45 min/18 sessions)	CTE/UC
Tong (2017)	China	40/31	41.9(4.2)	12	–	VAS	Tai Chi (30 min/36 sessions)	CTE
He (2013)	China	7/35	59.0(4.1)	12	–	VAS	Tai Chi (60 min/36 sessions)	CTE/NT
Wang et al. (2018)	China	45/32	45.2(15.0)	10	–	VAS/ODI	Qigong (5 min/50 sessions)	CTE
Chen (2020)	China	37/28	56(5.4)	4	–	VAS/ODI	Qigong (15 min/40 sessions)	CTE
Yao et al. (2020)	China	58/14	53.5(14.9)	24	–	VAS	Qigong (60 min/96 sessions)	CTE
Ding and Wang (2014)	China	20/20	61(4.7)	12	–	VAS	Qigong (40 min/60 sessions)	NT
Ning et al. (2015)	China	43/37	41.5(11.2)	12	–	VAS/ODI	Qigong (30 min/36 sessions)	CTE
Wu (2016)	China	42/36	39(7.6)	12	12	VAS/ODI	Qigong (30–40 min/36 sessions)	CTE
Phattharasupharerk et al. (2019)	Thailand	46/26	35.3(4.0)	6	–	VAS/RMDQ	Qigong (60 min/6 sessions)	NT
Teut et al. (2016)	Germany	156/20	72.7(5.7)	12	24	VAS	Qigong (90 min/12 sessions) Yoga (45 min/24 sessions)	NT
Blödt et al. (2015)	Germany	102/25	46.7(10.4)	12	24	VAS/RMDQ	Qigong (90 min/12 sessions)	CTE
Cruz-Díaz et al. (2018)	Spain	41/21	36.8(7.5)	12	–	VAS/RMDQ	Pilates (50 min/24 sessions)	NT
Valenza et al. (2017)	Spain	41/13	39(14)	8	–	VAS/RMDQ/ODI	Pilates (45 min/16 sessions)	UC
Kofotolis et al. (2016)	Spain	101/0	40.9(8.0)	8	–	RMDQ	Pilates (–/24 sessions)	UC/NT
Natour et al. (2015)	Brazil	47/13	47.9(12.1)	12	24	VAS/RMDQ	Pilates (50 min/24 sessions)	UC
Patti et al. (2016)	Italy	38	41.5(12.0)	14	28	ODI	Pilates (50 min/42 sessions)	CTE
Wajswelner et al. (2012)	Australia	48/39	49.1(15.2)	6	12–24	NRS	Pilates (60 min/12 sessions)	CTE
Mazloum et al. (2018)	Iran	47	39.6(9.3)	6	10	VAS/ODI	Pilates (50 min/18 sessions)	NT
Lee et al. (2014)	Korea	25	43.3(7.5)	12	–	VAS	Yoga (60 min/36 sessions)	NT
Kim et al. (2014)	Korea	30/0	Yoga group: 44.33/ Control group: 50.46	4	–	VAS/RMDQ/ODI	Yoga (30 min/12 sessions)	CTE
Neyaz et al. (2019)	India	35/35	35.5(12.4)	6	12	DVPRS/RMDQ	Yoga (35 min/6 sessions)	CTE
Tekur et al. (2012)	India	36/44	48.5(3.8)	1	–	VAS	Yoga (480 min/6 sessions)	CTE
Sherman et al. (2011)	USA	146/82	48.4(9.8)	12	26	RMDQ	Yoga (45–50 min/12 sessions)	UC
Cox et al. (2010)	UK	7/13	45	12	–	RMDQ	Yoga (75 min/12 sessions)	UC
Williams et al. (2009)	USA	69/21	48(11.1)	24	48	VAS/ODI	Yoga (90 min/48 sessions)	NT
Demirel et al. (2019)	Turkey	62/15	44.9(10.5)	6	–	VAS/ODI	Yoga (60 min/18 sessions)	CTE
Nambi et al. (2014)	India	32/28	44(9.0)	4	24	VAS	Yoga (60 min/4 sessions)	CTE
Bai et al. (2020)	China	60/0	33.3(2.5)	12	36	VAS	Yoga (75 min/36 sessions)	UC
Kuvačić et al. (2018)	Croatia	14/16	34.2(4.5)	8	–	NRS/ODI	Yoga (75 min/16 sessions)	UC
Saper et al. (2017)	USA	204/116	46(10.7)	12	26/40/52	NRS/RMDQ	Yoga (75 min/12 sessions)	CTE/ UC
Williams et al. (2005)	USA	30/14	48.3(7.2)	16	–	VAS	Yoga (30 min/16 sessions)	UC
Michalsen et al. (2021)	Germany	187/87	54.6(11.3)	8	–	VAS/RMDQ	Yoga (75 min/8 sessions)	CTE

M, male; F, female; VAS, visual numerical scale; NRS, numerical pain scale; BPI, Brief Pain Inventory; DVPRS, Defense and Veterans Pain Rating Scale; RMDQ, Roland Morris Disability Questionnaire; ODI, Oswestry Disability Index; UC, Usual care; NT, No treatment; CTE, Conventional therapeutic exercises.

### Quality appraisal of literature

**Figure 2:**
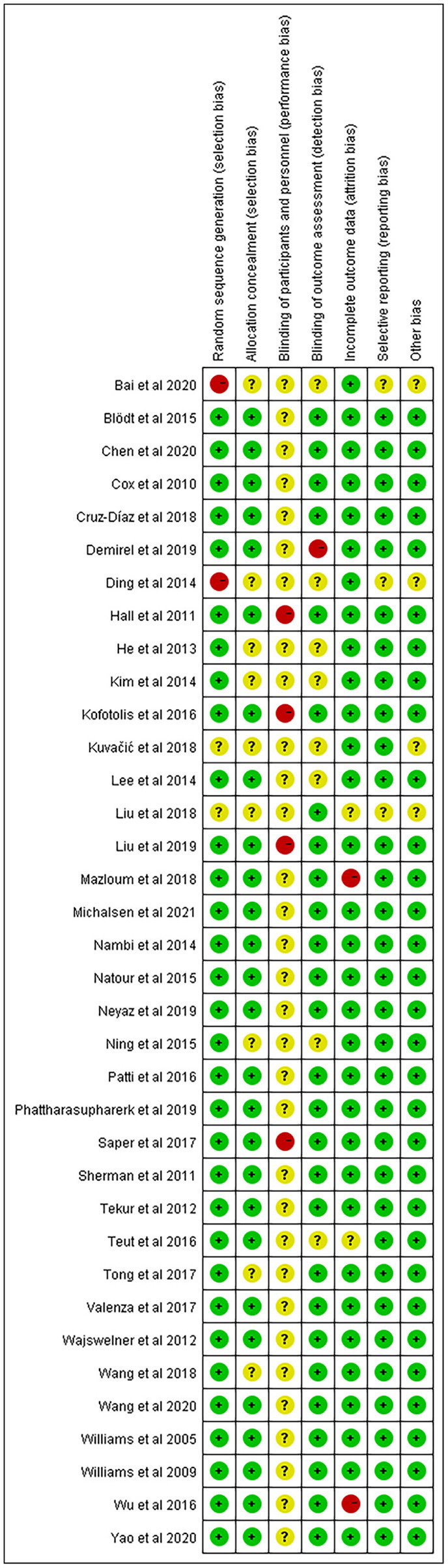
Percentage of studies examining the efficacy of mind-body exercise in patients with non-specific chronic low back pain with low, unclear and high risk of bias for each feature of the Cochrane Risk of Bias Tool.

**Figure 3:**
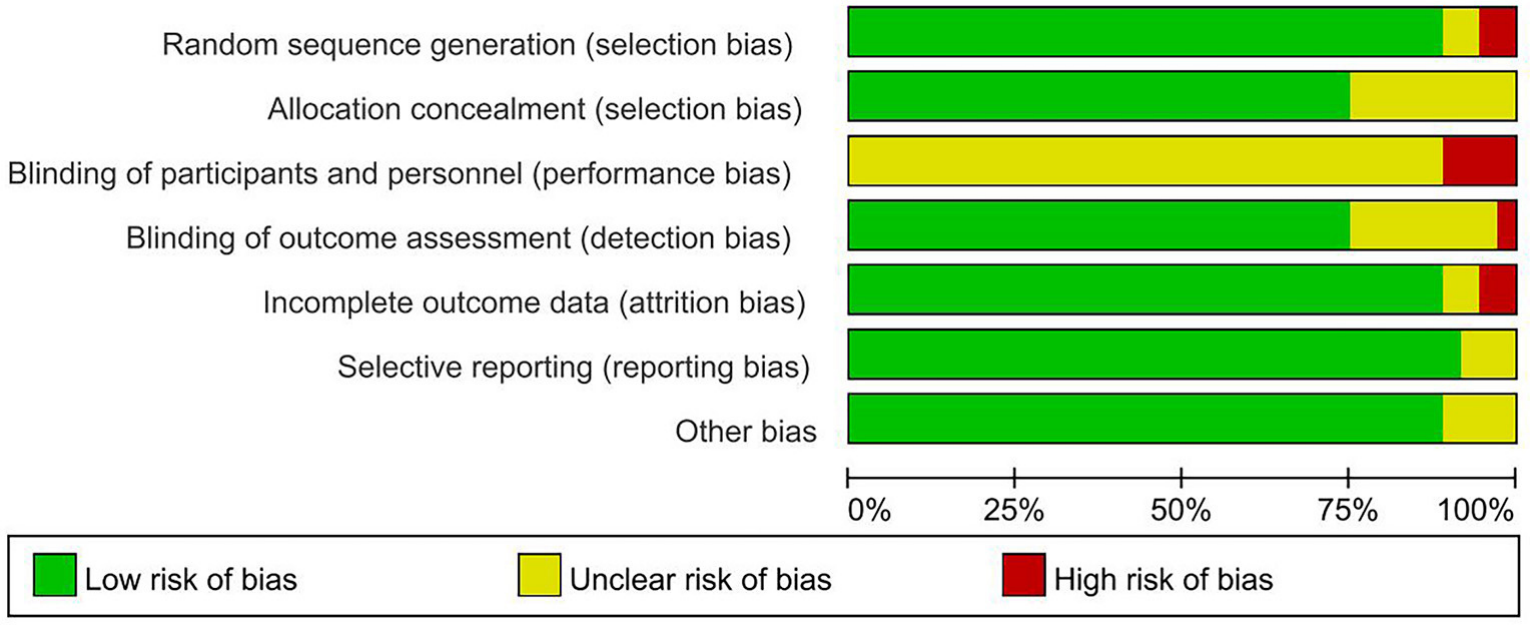
Methodological quality summary: Review authors' judgments about each methodological quality item for each included study.

The results of the Cochrane risk of bias assessment for each study were shown in Figures 2, 3. Due to insufficient random sequence generation, such as randomly assigning participants to groups based on their birth dates or hospitalization dates, two studies were classified as high risk. Owing to the MBE training involved in this trial, it was simply not able to blind the subjects to the treatment allocation. Therefore, subjects' blindness was considered to be a higher risk of bias in all studies. All studies were defined as unclear risk of bias, except those that explicitly stated that the subjects were not successfully blinded. One study was classified as high risk of bias because it did not utilize the appropriate blinding method for the evaluator. Meanwhile, two trials were defined as high-risk bias because of incomplete outcome data because of the high dropout rate of subjects or the number of subjects who left the group greatly varied between groups.

### Pairwise meta-analysis

We performed pairwise meta-analysis to compare the two interventions with pooled effect sizes. In terms of pain intensity, thirteen direct comparisons were performed to use a random effect model. TC was more efficacious than usual care (three RCTs; SMD: −1.29, 95% CI: −2.16 to −0.41; *I*^2^ ≥ 50%), and no treatment (two RCTs; SMD: −2.86, 95% CI: −3.65 to −2.07; *I*^2^ < 50%). Compared with usual care, yoga (five RCTs; SMD: −0.9, 95% CI: −1.51 to −0.28; *I*^2^ ≥ 50%) was more effective in decreasing pain intensity scores but Pilates (three RCTs; SMD: −1.85, 95% CI: −3.87 to 0.18; *I*^2^ ≥ 50%) and qigong (one RCTs; SMD: −0.32, 95% CI: −0.69 to 0.04) did not show a significant difference.

In terms of physical function, ten direct comparisons were constructed. Yoga was more efficacious than usual care (five RCTs; SMD: −1.45, 95% CI: −0.75 to −0.15; *I*^2^ < 50%), and no treatment (two RCTs; SMD: −1, 95% CI: −1.45 to −0.54; *I*^2^ < 50%). There were no differences in physical function score between usual care and Pilates, and TC. Supplementary Table 1 showed additional results of the pairwise meta-analysis and heterogeneity estimates.

### Network meta-analysis

**Figure 4:**
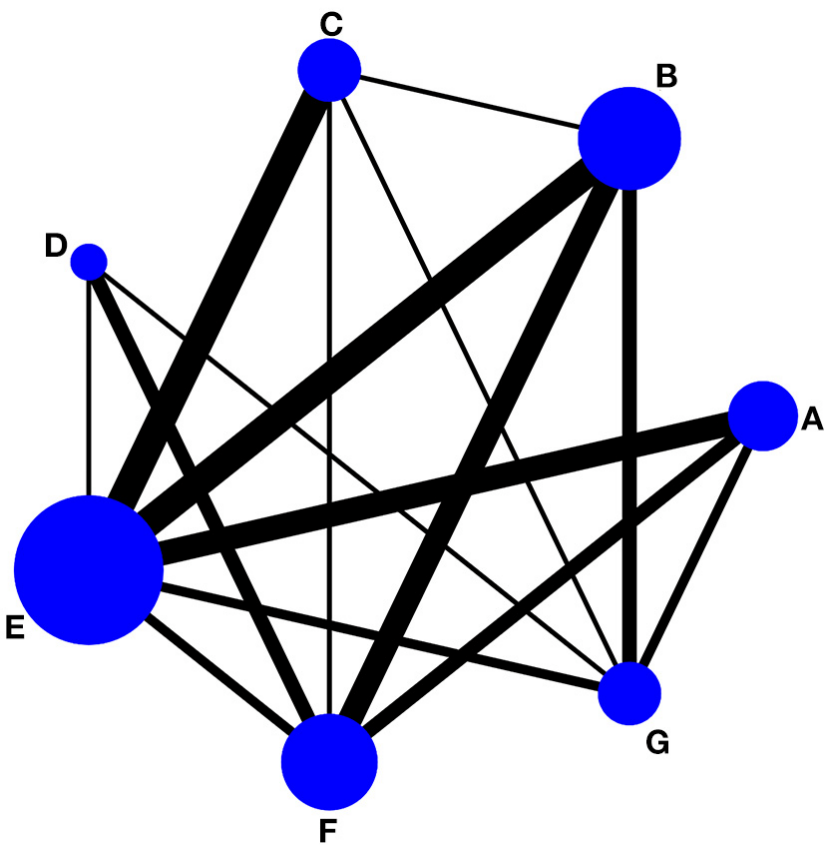
Network of evidence of pain intensity and the size of the nodes relates to the number of participants in that intervention type and the thickness of lines between interventions relates to the number of studies for that comparison. **(A)** tai chi, **(B)** yoga, **(C)** qigong, **(D)** Pilates, **(E)** control group (conventional therapeutic exercises), **(F)** control group (usual care), **(G)** control group (no treatment).

**Figure 5:**
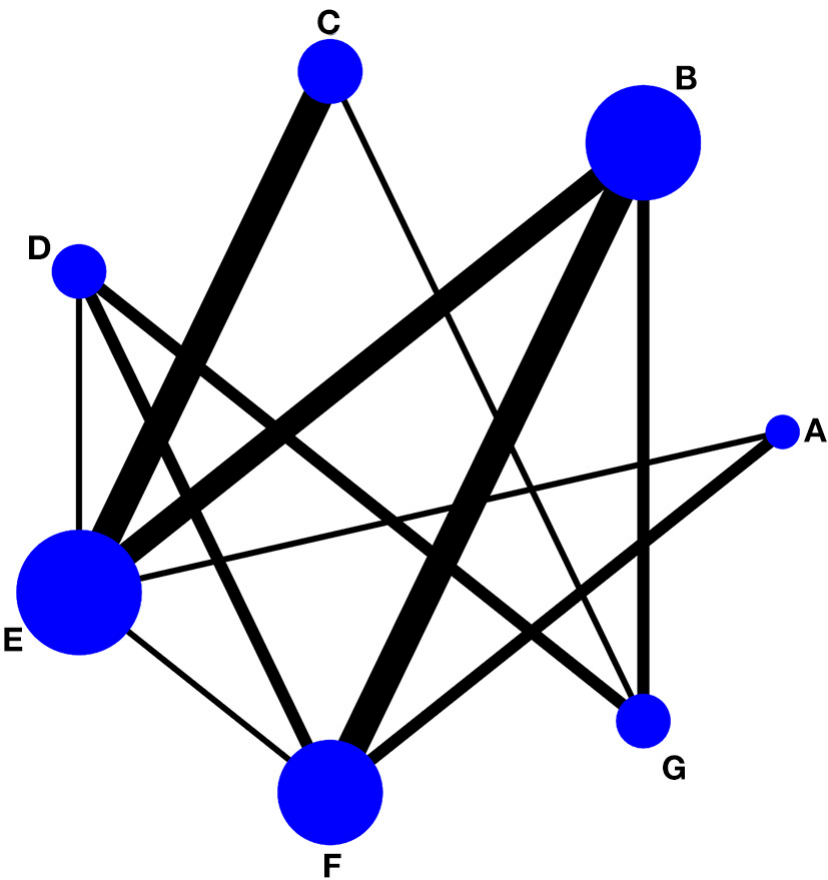
Network of evidence of physical function and the size of the nodes relates to the number of participants in that intervention type and the thickness of lines between interventions relates to the number of studies for that comparison. **(A)** tai chi, **(B)** yoga, **(C)** qigong, **(D)** Pilates, **(E)** control group (conventional therapeutic exercises), **(F)** control group (usual care), **(G)** control group (no treatment).

Figures 4, 5 showed the NMA figure for different interventions.

#### Pain intensity

**Figure 6:**
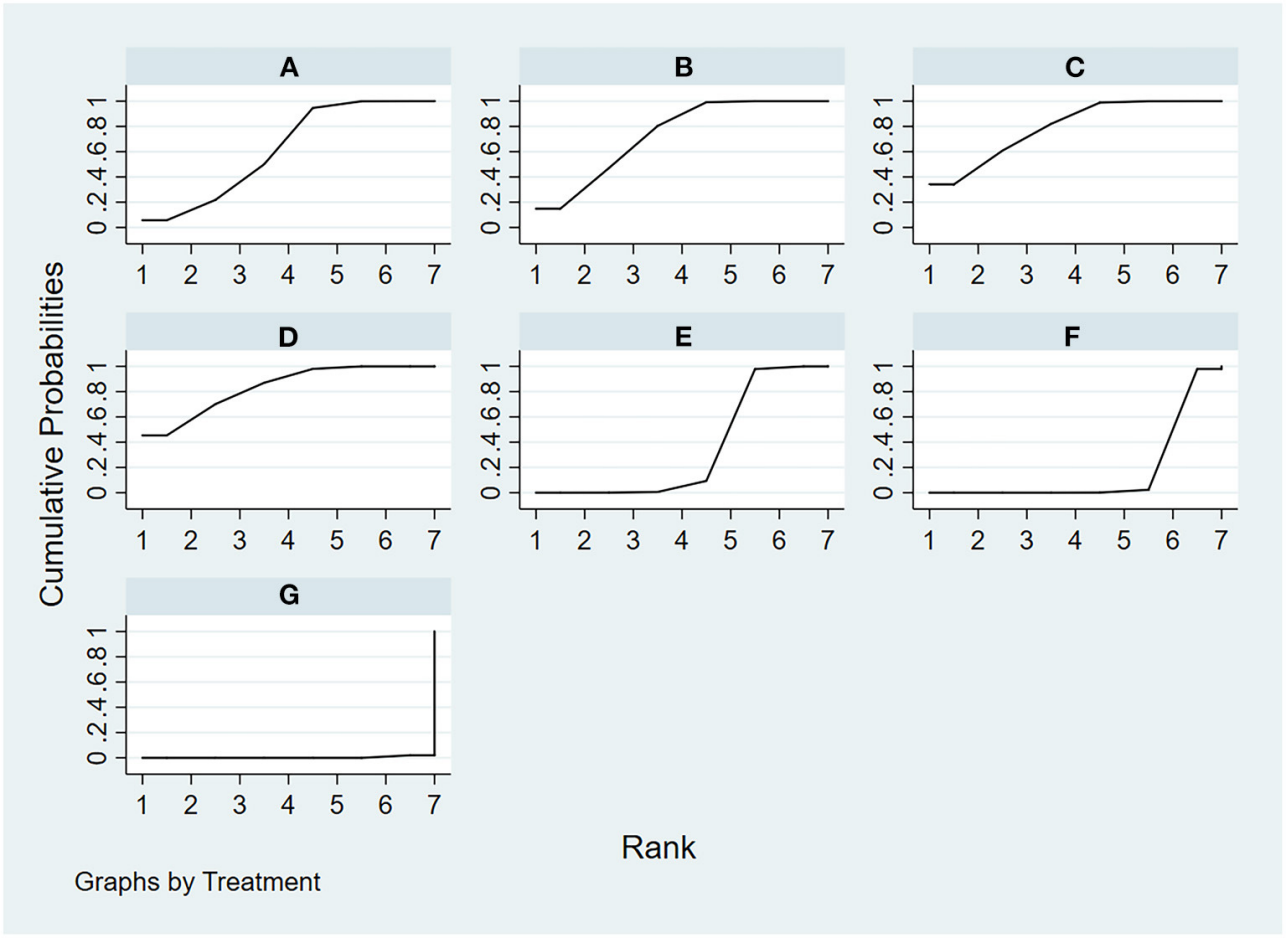
The rank probability of pain intensity various interventions based on the SUCRA. The SUCRA metric was used to rank the effectiveness of each treatment and identify the best treatment. **(A)** tai chi, **(B)** yoga, **(C)** qigong, **(D)** Pilates, **(E)** control group (conventional therapeutic exercises), **(F)** control group (usual care), **(G)** control group (no treatment).

There are a total of 32 included studies that evaluated pain intensity as presented in Figure 4 (The size of the circle represents the number of participants, and the thickness of the edge corresponds to the number of studies). The results of the node-splitting method reported that indirect and direct comparisons between each segmentation node were not statistically significantly different (*P* > 0.05), which indicated that the effect of consistency between studies was acceptable (see Supplementary Table 2). In terms of pain intensity improvement, the results of consistency NMA showed that compared to the control group with usual care (no exercise), Pilates intervention (SMD: −1.57, 95% CI: −2.44 to −0.71), TC intervention (SMD: −1.34, 95% CI: −2.15 to −0.53), yoga intervention (SMD: −1.18, 95% CI: −1.82 to −0.54), and qigong (SMD: −1.13, 95% CI: −1.97 to −0.29) were superior to the control group, the details of which are presented in Table 2. The ranking probability results of different MBE modes in terms of improving pain intensity indicated that Pilates (SUCRA = 86.6%) and TC (SUCRA = 77.2%) were among the best MBE interventions for pain. The control group with no treatment was most probably going to be the most ineffective (SUCRA = 0.6%). See Figure 6 for further details.

**Table 2:** League table on pain intensity.

**_D_**	**_A_**	**_B_**	**_C_**	**_E_**	**_F_**	**_G_**
D	0.23 (−0.84, 1.31)	0.39 (−0.57, 1.35)	0.44 (−0.63, 1.52)	0.85 (−0.09, 1.79)	1.57 (0.71, 2.44)	2.37 (1.32, 3.41)
−0.23 (−1.31, 0.84)	A	0.16 (−0.67, 0.99)	0.21 (−0.71, 1.13)	0.62 (−0.11, 1.35)	1.34 (0.53, 2.15)	2.13 (1.21, 3.06)
−0.39 (−1.35, 0.57)	−0.16 (−0.99, 0.67)	B	0.05 (−0.71, 0.82)	0.46 (−0.11, 1.02)	1.18 (0.54, 1.82)	1.97 (1.21, 2.74)
−0.44 (−1.52, 0.63)	−0.21 (−1.13, 0.71)	−0.05 (−0.82, 0.71)	C	0.41 (−0.22, 1.03)	1.13 (0.29, 1.97)	1.92 (1.03, 2.81)
−0.85 (−1.79, 0.09)	−0.62 (−1.35, 0.11)	−0.46 (−1.02, 0.11)	−0.41 (−1.03, 0.22)	E	0.72 (0.05, 1.40)	1.52 (0.75, 2.29)
**−1.57 (−2.44**, **−0.71)**	**−1.34 (−2.15**, **−0.53)**	**−1.18 (−1.82**, **−0.54)**	**−1.13 (−1.97**, **−0.29)**	**−0.72 (−1.40**, **−0.05)**	F	0.79 (−0.08, 1.66)
**−2.37 (−3.41**, **−1.32)**	**−2.13 (−3.06**, **−1.21)**	**−1.97 (−2.74**, **−1.21)**	**−1.92 (−2.81**, **−1.03)**	**−1.52 (−2.29**, **−0.75)**	−0.79 (−1.66, 0.08)	G

A, tai chi; B, yoga; C, qigong; D, Pilates; E, control group (conventional therapeutic exercises); F, control group (usual care); G, control group (no treatment). The bold font indicates a statistical difference.

#### Physical function

**Figure 7:**
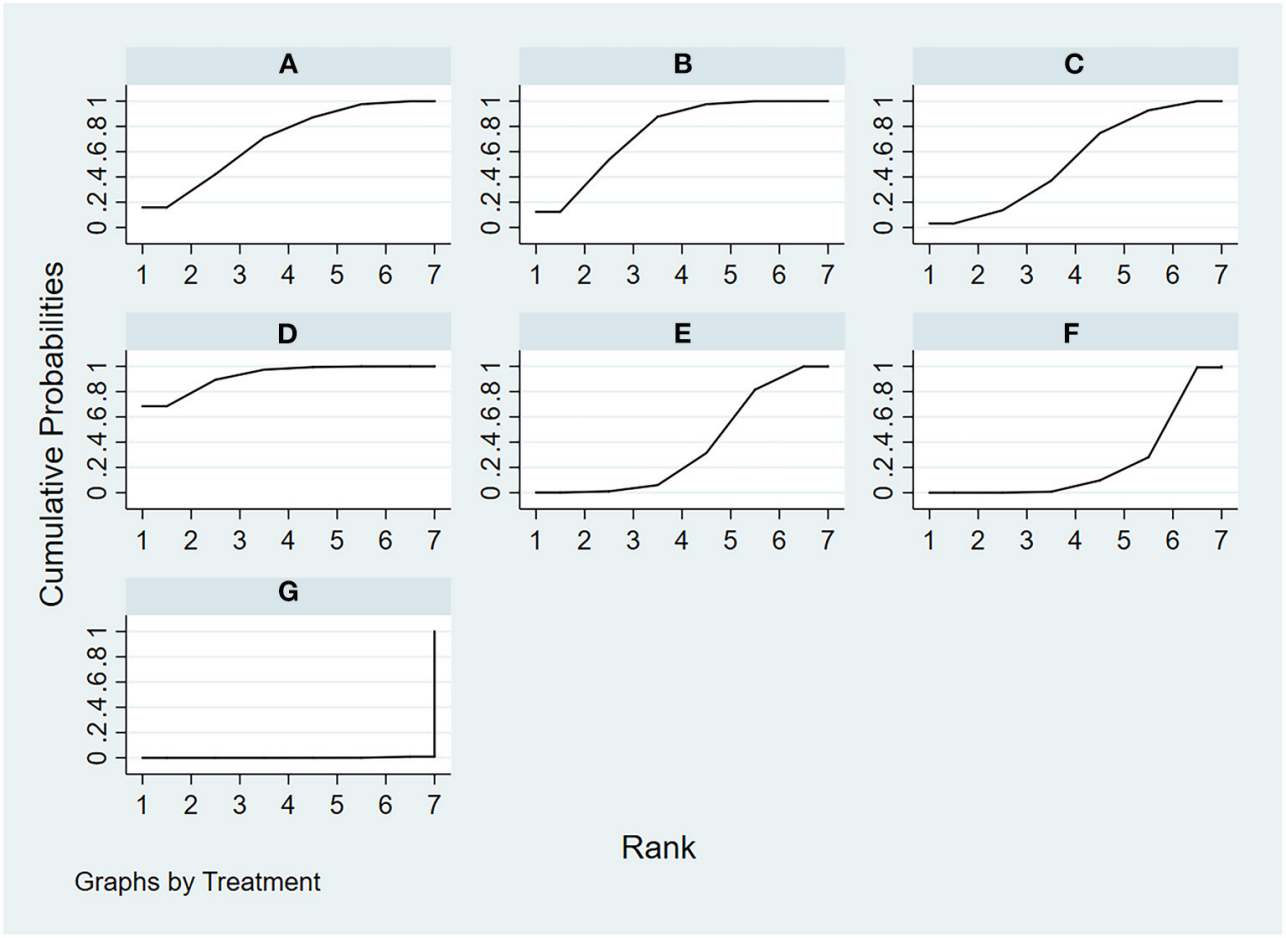
The rank probability of physical function various interventions based on the SUCRA. The SUCRA metric was used to rank the effectiveness of each treatment and identify the best treatment. **(A)** tai chi, **(B)** yoga, **(C)** qigong, **(D)** Pilates, **(E)** control group (conventional therapeutic exercises), **(F)** control group (usual care), **(G)** control group (no treatment).

A total of 24 included studies evaluated physical function outcomes, as shown in Figure 5. There was no evidence of inconsistency in the network (*P* > 0.05, see also Supplementary Table 3). In terms of physical function improvement, the results of consistency NMA showed that compared to the control group with usual care (no exercise), Pilates intervention (SMD: −1.68, 95% CI: −2.50 to −0.86), and yoga intervention (SMD: −0.63, 95% CI: −1.21 to −0.05) were superior to the control group; relative to the Qigong intervention group, Pilates intervention (SMD: −1.05, 95% CI: −1.89 to −0.21) was better than the qigong group in improving physical function, the details are shown in Table 3. The ranking probability results of different MBE modes in terms of improving physical function were initially positioned in the SUCRA for Pilates (SUCRA = 98.4%). The control group with no treatment was most probably going to be the most ineffective (SUCRA = 12.9%). See Figure 7 for further details.

**Table 3:** League table on physical function.

**_D_**	**_C_**	**_A_**	**_B_**	**_E_**	**_F_**	**_G_**
D	0.99 (−0.00, 1.98)	0.98 (−0.25, 2.21)	1.05 (0.21, 1.89)	1.20 (0.34, 2.06)	1.68 (0.86, 2.50)	1.73 (0.90, 2.56)
−0.99 (−1.98, 0.00)	C	−0.01 (−1.24, 1.23)	0.06 (−0.75, 0.86)	0.21 (−0.39, 0.82)	0.69 (−0.21, 1.59)	0.74 (−0.17, 1.66)
−0.98 (−2.21, 0.25)	0.01 (−1.23, 1.24)	A	0.06 (−1.02, 1.15)	0.22 (−0.88, 1.32)	0.70 (−0.30, 1.69)	0.75 (−0.51, 2.01)
**−1.05 (−1.89**, **−0.21)**	−0.06 (−0.86, 0.75)	−0.06 (−1.15, 1.02)	B	0.15 (−0.45, 0.76)	0.63 (0.05, 1.21)	0.68 (−0.10, 1.47)
−1.20 (−2.06, −0.34)	−0.21 (−0.82, 0.39)	−0.22 (−1.32, 0.88)	−0.15 (−0.76, 0.45)	E	0.48 (−0.25, 1.20)	0.53 (−0.31, 1.37)
**−1.68 (−2.50**, **−0.86)**	−0.69 (−1.59, 0.21)	−0.70 (−1.69, 0.30)	**−0.63 (−1.21**, **−0.05)**	−0.48 (−1.20, 0.25)	F	0.05 (−0.83, 0.93)
**−1.73 (−2.56**, **−0.90)**	−0.74 (−1.66, 0.17)	−0.75 (−2.01, 0.51)	−0.68 (−1.47, 0.10)	−0.53 (−1.37, 0.31)	−0.05 (−0.93, 0.83)	G

A, tai chi; B, yoga; C, qigong; D, Pilates; E, control group (conventional therapeutic exercises); F, control group (usual care); G, control group (no treatment). The bold font indicates a statistical difference.

### Sensitivity analysis

We tested the sensitivity analysis of the results of NMA by comparing the results of the random effect model with the fixed effect model, and found no significant difference between the results obtained using the two models, which indicated that our results were robust.

### Publication bias

**Figure 8:**
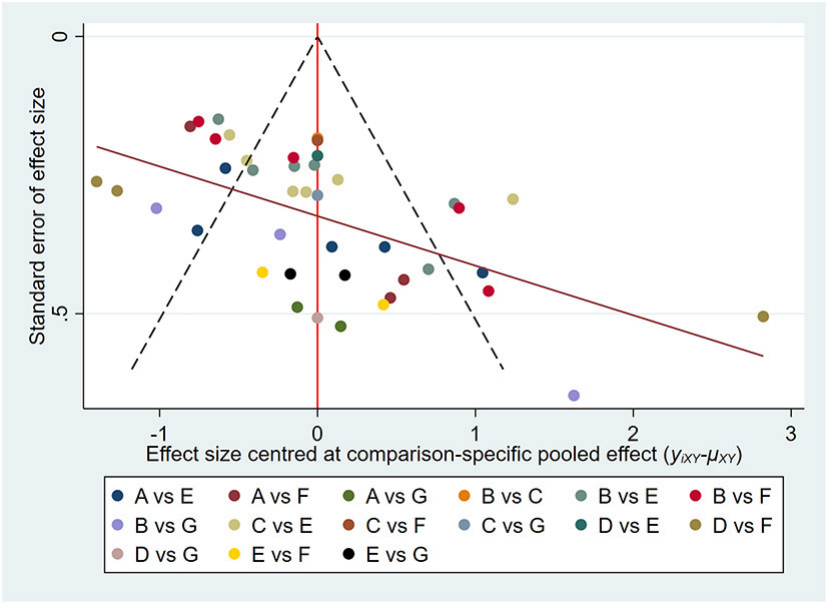
Pain intensity: Funnel plot showing the publication bias of the included randomized controlled trials. The red line represents the null hypothesis that independent effect size estimates do not differ from the comparison-specific pooled estimates. **(A)** tai chi, **(B)** yoga, **(C)** qigong, **(D)** Pilates, **(E)** control group (conventional therapeutic exercises), **(F)** control group (usual care), **(G)** control group (no treatment).

**Figure 9:**
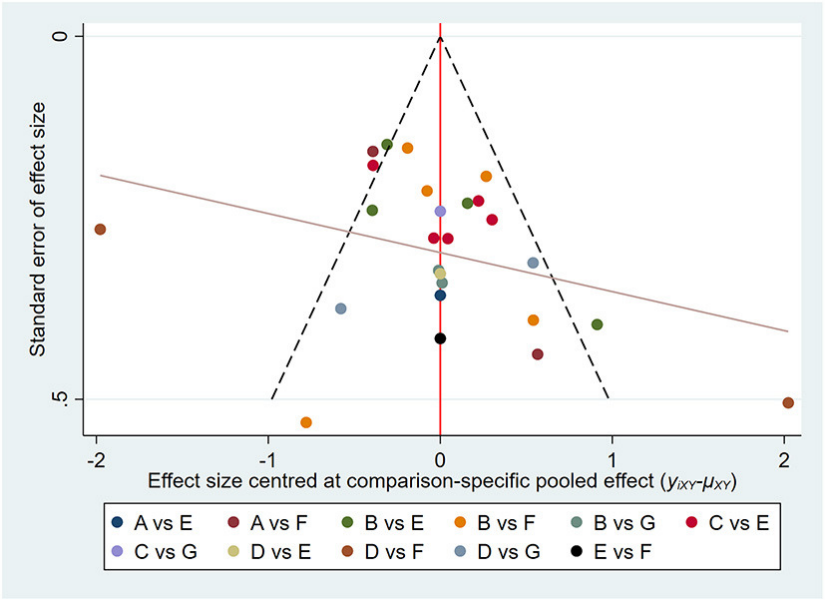
Physical function: Funnel plot showing the publication bias of the included randomized controlled trials. The red line represents the null hypothesis that independent effect size estimates do not differ from the comparison-specific pooled estimates. **(A)** tai chi, **(B)** yoga, **(C)** qigong, **(D)** Pilates, **(E)** control group (conventional therapeutic exercises), **(F)** control group (usual care), **(G)** control group (no treatment).

We built and assessed a modified funnel plot to detect possible publication bias for all indicators. The findings reveal that the majority of points are evenly distributed along both sides of the midline and are primarily focused there. This indicates that our results are robust and there is no significant publication offset. See Figures 8, 9 for further details.

## Discussion

In our study, we tried to compare the curative effect of the four most popular MBE modes in improving pain intensity and physical function to identify optimal MBE interventions for patients with CNLBP. Pilates might be the best MBE mode for decreasing pain intensity, followed by TC, yoga, and qigong. However, the differences were minor for yoga and qigong. Pilates continued to be the best performer in improving physical function, with little difference in the remaining three modes. Interestingly, TC performed well in managing the pain but significantly less well than Pilates in improving physical function. Overall, Pilates is perhaps the most appropriate MBE intervention for treating patients with CNLBP.

Our NMA found that Pilates was the most effective mode in decreasing pain intensity, consistent with prior reviews on other exercise therapies (Owen et al., 2020). There was a close correlation between CNLBP and core muscles, particularly deep multifidus and transversus abdominis (Ferreira et al., 2010). In CNLBP patients, activation of multifidus psoas and transverse abdominis is delayed or reduced, and physiologic tonic activation of transverse abdominis is lost during gait and extremity movement. In addition, dysfunction of these muscles might lead to the loss of lumbar support and increase the stress and load on the joints and ligaments of the spine (Ferreira et al., 2010; Hides et al., 2011). This may cause pain and functional abnormalities in CNLBP patients, thus, improving core functions is the key to treating CNLBP (Tang et al., 2016). Developed by Joseph H. Pilates, Pilates exercise therapy is used to improve an individual's “flexibility, strength, and body awareness” and it is referred to as a technique that focuses on core stability, posture, breathing, flexibility, strength, and muscle control (Wells et al., 2012). Moreover, the Pilates approach focuses on strengthening the lumbar region with the active involvement of the core muscles (Rydeard et al., 2006). Previous studies comparing core muscle activation in three different postures between Pilates practitioners and the general population have found that the core muscle activation in Pilates practitioners is significantly higher than that in the general population (Lee, 2021). Therefore, Pilates may decrease pain intensity by enhancing the core muscle. Although current evidence shows the analgesic effect of Pilates in patients with CNLBP, objective neurophysiological studies to elucidate the analgesic mechanism are lacking. Widespread oscillatory abnormalities in chronic pain patients and enhanced alpha activity by therapeutic means are associated with pain relief (Arendsen et al., 2018; Ahn et al., 2019). Bian et al. (2013) found that peak alpha power increased for healthy participants during Pilates training, which indicates that Pilates practice may relieve pain by modulating peak alpha frequency in chronic pain patients. Future studies may consider exploring the effect of Pilates training on peak alpha frequency in patients with CNLBP to further clarify the neurophysiological mechanism of Pilates analgesia. Apart from that, Pilates has the advantage that the exercises can be performed in various settings, with or without equipment, thereby keeping the spine in a neutral position and avoiding excessive impact or stress on muscles, joints, and tissues as compared with other MBE modes. As the exercises progress and an individual wishes to increase the difficulty of the activities performed, one can incorporate the use of various types of equipment, including the reformer, cadillac, ladder barrel, and step chair.

A novel finding from this NMA is that TC (SUCRA = 77.2%) may be the intervention that came closest to the effect of Pilates (SUCRA = 86.6%) in reducing pain intensity among the other three MBE modes. Meanwhile, TC originating in China is an established form of gentle MBE mode and incorporates physical, psychosocial, spiritual, and behavioral elements to improve physical and mental health (Wang et al., 2018). Although the underlying mechanism of TC remains unclear, the effect of TC may be attributable to the potential of these exercises to influence altered central elements. Furthermore, when practicing TC, the body's center of gravity constantly changes with the movements, the spine is in an unstable state, and the central nervous system recruits more muscle fibers to maintain stability, which strengthens the core muscles to some extent. Respiratory exercise therapy does have a therapeutic effect on patients with low back pain (Mehling et al., 2005). Herein, TC emphasizes reverse abdominal breathing, which strengthens the core muscles during the breathing process. Notably, CNLBP is often accompanied by structural and functional connectivity abnormalities in brain regions (Ji and Neugebauer, 2011; Neugebauer et al., 2020). Regular TC practice can bring about regional structural changes in the precentral gyrus, insular sulcus, and middle frontal sulcus (Wei et al., 2013). A previous RCT also found moderate to high correlations between TC-associated pre-post differences in the functional connectivity of the amygdala-medial prefrontal cortex (Shen et al., 2021). Therefore, TC may directly affect the cerebral cortex to regulate pain through regular practice. Considering that it is not too difficult, cost-effective, and safe, TC and qigong are often chosen by elderly people to practice (Li et al., 2020; Siu et al., 2021). Pilates and yoga are more difficult than TC and qigong to practice and usually require the guidance of a professional instructor to ensure safety during practice (Achilefu et al., 2017; Zou et al., 2019). Therefore, TC and qigong seem to be worthy of promotion in the elderly population with CNLBP.

Guidelines about CNLBP suggest that treatment should pay more attention to improving pain intensity and its associated dysfunction (Oliveira et al., 2018). Our study suggested that Pilates (SUCRA = 98.4%) had the highest probability of improving physical function. Interestingly, TC was effective in reducing pain intensity, however, lagged far behind Pilates in improving physical function. Age is an essential factor affecting physical function (Maher et al., 2017). We compared the groups included in this study, and found that the average age of the Pilates group was younger than that of the TC group. It may be the reason why the function improvement effect of the Pilates group was better than that of the TC group. Therefore, it would be interesting to see if Pilates outperforms other MBE modes under strict age restrictions. There are various schools of TC in China, such as Yang-style TC and Chen-style TC. Although they are all based on the basic theories of the balance of Yin and Yang, the balance of the five elements, and the interaction between man and nature (Peng, 2012; Zhang et al., 2019), there are still great differences in movement characteristics and the degree of difficulty. Among the included studies, there are three that used Chen-style TC, one article designed an improved TC movement for CNLBP, and the other two did not tell, which may be partly responsible for the poor effect of TC in improving function. Similar to TC, there are various kinds of qigong, such as Neiyanggong (Blödt et al., 2015) and Wuqinxi (Yao et al., 2020). Different kinds of qigong have different effects on the physical function of CNLBP patients. A meta-analysis (Bai et al., 2015) involving 10 RCTs indicated that only internal qigong could improve chronic pain in adults. Therefore, it is a meaningful research direction to explore which style of TC or qigong movements are most suitable for enhancing physical function for patients with CNLBP.

### Implication

Broadly, our NMA found Pilates may be the most recommended MBE mode for patients with CNLBP. As compared with previous studies (Owen et al., 2020; Hayden et al., 2021b; Fernández-Rodríguez et al., 2022), the results all agree that Pilates is best in terms of decreasing pain intensity and improving physical function. However, the difference is that our study included TC and qigong, which are often overlooked by previous studies. The findings suggest that TC is comparable to Pilates in decreasing pain intensity, which provides a new option for managing pain in patients with CNLBP.

### Strengths and limitations

To our knowledge, this NMA is the first to compare the effects of different MBE modes in CNLBP. It explores a comprehensive ranking of four popular MBE treatments, thereby identifying the best options for improving pain intensity and physical function in CNLBP patients. Our searches were not limited by publication date or language, and included Chinese databases and gray literature. Given that TC and qigong originated in China, various high-quality studies have been published in Chinese journals, thus making our review more comprehensive.

Following are the limitations of our study. First, it is unable to blind subjects during an MBE intervention, which may lead to a potential risk of performance bias. However, this is an inherent limitation of such studies, usually reported in meta-analyses of exercise programs (Goh et al., 2019; Zou et al., 2019; Owen et al., 2020). Second, our review did not include psychology-related dependent variables such as depression, which is an important indicator for evaluating the success of CNLBP treatment. Based on several previous studies (Tekur et al., 2012; Park et al., 2020), MBE has reported positive results in treating psychological distress, such as depression and anxiety in patients with LBP. However, only six of our included studies reported depression-related results (Williams et al., 2009; Tekur et al., 2012; Lee et al., 2014; Teut et al., 2016; Kuvačić et al., 2018; Wang, 2018). Therefore, future studies should be considered to further explore the effect of MBE on psychological distress in CNLBP patients and the underlying mechanisms. Finally, because of the small number of studies and limited evidence for direct comparisons of interventions, readers should view these findings with caution. Therefore, it also emphasizes the need to further expand related research.

## Conclusions

Our NMA shows that Pilates might be the best MBE therapy for the non-pharmacologic treatment of CNLBP in pain intensity and physical function. It has a reasonable benefit, which would be a powerful option for patients who don't profit from existing pharmacological medicines. Our study provides richer options for CNLBP management and more evidence for MBE treatment of CNLBP. However, more high-quality, large-sample, multicenter, long-term follow-up RCTs directly compare the efficacy of two or more MBE modes in patients with CNLBP to further confirm our findings.

## Data availability statement

The original contributions presented in the study are included in the article/Supplementary material, further inquiries can be directed to the corresponding author/s.

## Author contributions

JS and Z-YH wrote the manuscript. Y-FW, Z-YH, JS, and Y-LW contributed to the conception. Z-YH and H-ZZ searched the literature. JS and Y-RW were involved in the data analysis. Y-YL, JS, Z-YH, Y-TL, and Y-LW contributed to the acquisition of data. All authors contributed to the article and approved the submitted version.

## Funding

This research was funded by the Guangdong Hopson-Pearl River Education Development Foundation, grant number H20190116202012724.

## Conflict of interest

The authors declare that the research was conducted in the absence of any commercial or financial relationships that could be construed as a potential conflict of interest.

## Publisher's note

All claims expressed in this article are solely those of the authors and do not necessarily represent those of their affiliated organizations, or those of the publisher, the editors and the reviewers. Any product that may be evaluated in this article, or claim that may be made by its manufacturer, is not guaranteed or endorsed by the publisher.
