# Supplementary material for: Optimal modes of mind-body exercise for treating chronic non-specific low back pain: Systematic review and network meta-analysis
Source: Front Neurosci. 2022 Nov 17;16:1046518. doi: 10.3389/fnins.2022.1046518 (PMC9713308; doi:10.3389/fnins.2022.1046518)
Supplement: Supplementary file 1 [file Table_1.docx]

**Supplementary Table S1. Results of Pairwise Meta-Analysis**

| Variable | Comparison | Number | SMD (95% CI) | I^2^ (%) | P |
| --- | --- | --- | --- | --- | --- |
| Pain intensity | A VS E | 5 | -0.51(-1.13, 0.11) | 75 | 0.003 |
|  | A VS F | 3 | -1.29(-2.16, -0.41) | 78 | 0.01 |
|  | A VS G | 2 | -2.86(-3.65, -2.07) | 0 | 0.36 |
|  | B VS C | 1 | -0.07(-0.43, 0.29) | - | - |
|  | B VS E | 6 | -0.77(-1.22, -0.32) | 81 | <0.0001 |
|  | B VS F | 5 | -0.9(-1.51, -0.28) | 89 | <0.0001 |
|  | B VS G | 3 | -1.61(-2.85, -0.37) | 86 | 0.0008 |
|  | C VS E | 6 | -0.48(-0.97, 0.01) | 84 | <0.0001 |
|  | C VS F | 1 | -0.32(-0.69, 0.04) | - | - |
|  | C VS G | 1 | -1.89(-2.45, -1.33) | - | - |
|  | D VS E | 1 | -0.21(-0.63, 0.21) | - | - |
|  | D VS F | 3 | -1.85(-3.87, 0.18) | 97 | <0.0001 |
|  | D VS G | 1 | -2.69(-3.68, -1.7) | - | - |
|  |  |  |  |  |  |
| Physical function | A VS E | 1 | -0.7(-1.4, 0.01) | - | - |
|  | A VS F | 2 | -0.52(-1.3, 0.27) | 67 | 0.08 |
|  | B VS E | 4 | -0.2(-0.64, 0.25) | 73 | 0.01 |
|  | B VS F | 5 | -0.45(-0.75, -0.15) | 48 | 0.11 |
|  | B VS G | 2 | -1(-1.45, -0.54) | 0 | 0.97 |
|  | C VS E | 5 | -0.22(-0.5, 0.06) | 44 | 0.13 |
|  | C VS G | 1 | -0.58(-1.05, -0.11) | - | - |
|  | D VS E | 1 | -0.33(-0.97,0.31) | - | - |
|  | D VS F | 2 | -2.65(-6.57,1.28) | 98 | <0.0001 |
|  | D VS G | 2 | -1.51(-2.60, -0.41) | 81 | 0.02 |
| **Note:** A: tai chi, B: yoga, C: qigong, D: Pilates, E: control group (conventional therapeutic exercises), F: control group (usual care), G: control group (no treatment) | | | | | |

**Supplementary Table S2. Consistency test for Pain intensity.**

| Side | Direct |  | Indirect |  | Difference | |  | tau |
| --- | --- | --- | --- | --- | --- | --- | --- | --- |
|  | Coef. | Std. Err. | Coef. | Std. Err. | Coef. | Std. Err. | P>z |  |
| A E | 0.4933797 | 0.4200489 | 1.114489 | 0.8368247 | -0.6211089 | 0.9330543 | 0.506 | 0.8687216 |
| A F | 1.41521 | 0.5488069 | 1.241843 | 0.6521709 | 0.173367 | 0.8525887 | 0.839 | 0.8744307 |
| A G | 2.82525 | 0.693901 | 1.566878 | 0.6241891 | 1.258372 | 0.9330929 | 0.177 | 0.8414302 |
| B C | -0.0737951 | 0.8958889 | 0.0813482 | 0.4447715 | -0.1551433 | 1.000178 | 0.877 | 0.8769075 |
| B E | 0.8018859 | 0.3585269 | -0.0887598 | 0.4507202 | 0.8906457 | 0.5759026 | 0.122 | 0.8353897 |
| B F | 0.9256865 | 0.4035117 | 1.664021 | 0.5560689 | -0.7383344 | 0.6864446 | 0.282 | 0.8579371 |
| B G | 1.570438 | 0.5547755 | 2.355225 | 0.5407808 | -0.7847867 | 0.7746239 | 0.311 | 0.8521042 |
| C E | 0.4920222 | 0.3715129 | 0.1371341 | 0.6611239 | 0.3548881 | 0.758399 | 0.64 | 0.8736934 |
| C F | 0.3207493 | 0.8786438 | 1.383648 | 0.4918755 | -1.062899 | 1.006967 | 0.291 | 0.8584451 |
| C G | 1.887295 | 0.921822 | 1.936782 | 0.5346588 | -0.0494873 | 1.065653 | 0.963 | 0.8761961 |
| D E | 0.2126586 | 0.8909843 | 1.116318 | 0.5735283 | -0.9036596 | 1.059617 | 0.394 | 0.8646358 |
| D F | 1.721064 | 0.5458518 | 1.293201 | 0.7700496 | 0.4278633 | 0.9427719 | 0.65 | 0.8730968 |
| D G | 2.686374 | 1.007039 | 2.241942 | 0.6394468 | 0.4444324 | 1.192904 | 0.709 | 0.8699149 |
| E F | 1.788243 | 0.6599083 | 0.3633534 | 0.3800064 | 1.42489 | 0.7601312 | 0.061 | 0.8160718 |
| E G | 1.572592 | 0.6883871 | 1.490106 | 0.4855431 | 0.0824859 | 0.8377524 | 0.922 | 0.8746346 |

| Side | Direct |  | Indirect |  | Difference | |  | tau |
| --- | --- | --- | --- | --- | --- | --- | --- | --- |
|  | Coef. | Std. Err. | Coef. | Std. Err. | Coef. | Std. Err. | P>z |  |
| A E | 0.661628 | 0.789196 | -0.25407 | 0.816837 | 0.915697 | 1.133872 | 0.419 | 0.704021 |
| A F * | 0.623078 | 0.552814 | 1.343104 | 1.643933 | -0.72003 | 1.745451 | 0.68 | 0.716043 |
| B E | 0.250296 | 0.383884 | -0.05589 | 0.568236 | 0.306191 | 0.68636 | 0.656 | 0.721275 |
| B F | 0.432231 | 0.342192 | 1.256049 | 0.607368 | -0.82382 | 0.697836 | 0.238 | 0.7008 |
| B G | 0.995156 | 0.553043 | 0.309932 | 0.604721 | 0.685223 | 0.819479 | 0.403 | 0.708576 |
| C E | 0.246201 | 0.340271 | -0.02209 | 0.924161 | 0.268296 | 0.984771 | 0.785 | 0.720464 |
| C G | 0.580796 | 0.759695 | 0.849117 | 0.62668 | -0.26832 | 0.984816 | 0.785 | 0.720467 |
| D E | 0.332849 | 0.753657 | 1.616154 | 0.52804 | -1.28331 | 0.920231 | 0.163 | 0.679022 |
| D F | 2.360354 | 0.549886 | 0.819798 | 0.597002 | 1.540557 | 0.81299 | 0.058 | 0.660309 |
| D G | 1.503522 | 0.562905 | 2.07847 | 0.69195 | -0.57495 | 0.89251 | 0.519 | 0.717855 |
| E F | 0.46948 | 0.831088 | 0.482931 | 0.431217 | -0.01345 | 0.939132 | 0.989 | 0.719901 |

**Supplementary Table S3. Consistency test for Physical function.**
