# Supplementary material for: Optimal modes of mind-body exercise for treating chronic non-specific low back pain: Systematic review and network meta-analysis
Source: Front Neurosci. 2022 Nov 17;16:1046518. doi: 10.3389/fnins.2022.1046518 (PMC9713308; doi:10.3389/fnins.2022.1046518)
Supplement: Supplementary file 2 [file Data_Sheet_1.docx]

**PubMed**

**#1** “Yoga”[MeSH Terms]OR”yoga” [Title/Abstract]OR ”hatha yoga” [Title/Abstract] OR “hot yoga” [Title/Abstract] OR “iyengar yoga” [Title/Abstract] OR “pranayama” [Title/Abstract] OR “yoga nidra” [Title/Abstract]

**#2** “tai ji"[Title/Abstract] OR "Tai Chi"[Title/Abstract] OR "chi tai"[Title/Abstract] OR "Tai Ji Quan"[Title/Abstract] OR "ji quan tai"[Title/Abstract] OR "quan tai ji"[Title/Abstract] OR "Taiji"[Title/Abstract] OR "Taijiquan"[Title/Abstract] OR "T'ai Chi"[Title/Abstract] OR "Tai Chi Chuan"[Title/Abstract] OR "tai ji"[MeSH Terms]

**#3** "qigong"[MeSH Terms] OR “Qi Gong” [Title/Abstract] OR “Ch'i Kung” [Title/Abstract] OR “baduanjin” [Title/Abstract] OR “yijinjing” [Title/Abstract] OR “liuzijue”[Title/Abstract] OR”wuqinxi” [Title/Abstract]

**#4** “mind-body” [Title/Abstract] OR “mind exercise” [Title/Abstract]

**#5**"Exercise Movement Techniques"[MeSH Terms] OR "pilates based exercises"[Title/Abstract] OR "pilates based exercises"[Title/Abstract] OR "Pilates Training"[Title/Abstract] OR "Pilates"[Title/Abstract]

**#6**: #1 OR #2 OR #3 OR #4OR#5

**#7** ”Low Back Pain” [MeSH Terms] OR “Back Pain” [Title/Abstract] OR “Low Back Pains” [Title/Abstract] OR “Pain, Low Back” [Title/Abstract] OR “Pains, Low Back” [Title/Abstract] OR “Lumbago” [Title/Abstract] OR “Lower Back Pain” [Title/Abstract] OR “Back Pain, Lower” [Title/Abstract] OR “Lower Back Pains” [Title/Abstract] OR “Pain, Lower Back” [Title/Abstract] OR “Pains, Lower Back” [Title/Abstract] OR “Low Back Ache” [Title/Abstract] OR “Ache, Low Back” [Title/Abstract] OR “Low Backache” [Title/Abstract] OR “Backache, Low” [Title/Abstract] OR “Low Back Pain, Postural” [Title/Abstract] OR “Postural Low Back Pain” [Title/Abstract] OR “Low Back Pain, Recurrent” [Title/Abstract] OR “Recurrent Low Back Pain” [Title/Abstract] OR “Low Back Pain, Mechanical” [Title/Abstract] OR “Mechanical Low Back Pain” [Title/Abstract]

**#8**: #6 AND #7

**Embase**

**#1** 'low back pain'/exp

**#2 '**low back pain':ti,ab,kw OR 'back pain':ti,ab,kw OR 'lower back pain':ti,ab,kw OR 'low back ache':ti,ab,kw OR 'lumbago':ti,ab,kw OR 'dorsalgia':ti,ab,kw OR backache:ti,ab,kw OR 'spondylosis':ti,ab,kw OR 'back disorder':ti,ab,kw

**#3**: #1 OR #2

**#4**: 'Tai Chi'/exp

**#5**: 'taiji quan':ti,ab,kw OR taijiquan:ti,ab,kw OR taiji:ti,ab,kw OR 'tai ji':ti,ab,kw OR 'tai chi':ti,ab,kw OR 'chi tai':ti,ab,kw

**#6**: 'qigong'/exp

**#7**: qigong:ti,ab,kw OR 'qi gong':ti,ab,kw OR qi‐gong:ti,ab,kw OR 'qi kung':ti,ab,kw OR 'jhi gong':ti,ab,kw OR 'chi gung':ti,ab,kw OR 'qi chung':ti,ab,kw OR 'chi kung':ti,ab,kw OR 'kung chi':ti,ab,kw OR baduanjin:ti,ab,kw OR wuqinxi:ti,ab,kw OR liuzijue:ti,ab,kw OR yijinjing:ti,ab,kw

**#8**: 'yoga'/exp

**#9:** yoga:ti,ab,kw OR 'hatha yoga':ti,ab,kw OR 'hot yoga':ti,ab,kw OR 'iyengar yoga':ti,ab,kw OR pranayama:ti,ab,kw OR 'yoga nidra':ti,ab,kw

**#10** 'mind -body exercise':ti,ab,kw OR 'mindful body':ti,ab,kw

**#11 'pilates'/exp**

**#12 'pilates based exercises':ti,ab,kw OR 'pilates training':ti,ab,kw OR 'pilates':ti,ab,kw**

**#13**: #4 OR #5 OR #6 OR #7 OR #8 OR #9 OR #10OR#11OR#12

**#14**: #3 AND#13

**Web of Science**

**#1**:”Low Back Pain” OR “Back Pain” OR “Low Back Pains” OR “Lumbago” OR “Lower Back Pain” OR “Lower Back Pains” OR “Low Back Ache” OR “Low Backache” OR “Postural Low Back Pain” OR “Recurrent Low Back Pain” OR “Mechanical Low Back Pain”

**#2** ”Yoga” OR “hatha yoga” OR “hot yoga” OR “iyengar yoga” OR “pranayama” OR “yoga nidra”

**#3** "tai ji" OR "Tai Chi" OR "chi tai" OR "Tai Ji Quan" OR "ji quan tai" OR "quan tai ji" OR "Taiji" OR "Taijiquan" OR "T'ai Chi" OR "Tai Chi Chuan"

**#4** "qigong" OR “Qi Gong” OR “Ch'i Kung” OR “baduanjin” OR “yijinjing” OR “liuzijue” OR”wuqinxi”

**#5** “mind-body” OR “mind exercise”

**#6**："pilates based exercises"OR "pilates based exercises" OR "Pilates Training"OR "Pilates"

**#7**: #2 OR #3 OR #4 OR #5OR#6

**#8**: #7AND#1

**Cochrane**

**#1** MeSH descriptor: [Low Back Pain] explode all trees

**#2** ("Low Back Pain" OR "Back Pain" OR "Low Back Pains" OR "Lumbago" OR "Lower Back Pain" OR "Lower Back Pains" OR "Low Back Ache" OR "Low Backache" OR "Postural Low Back Pain" OR "Recurrent Low Back Pain" OR "Mechanical Low Back Pain" OR "backache" OR "back pain"):ti,ab,kw(Word variations have been searched)

**#3**: #1 or #2

**#4** MeSH descriptor: [Tai Ji] explode all trees

**#5** ("tai ji" OR "Tai Chi" OR "chi tai" OR "Tai Ji Quan" OR "ji quan tai" OR "quan tai ji" OR Taiji OR "Taijiquan" OR "T'ai Chi" OR "Tai Chi Chuan"):ti,ab,kw(Word variations have been searched)

**#6**: #4 OR #5

**#7**: MeSH descriptor: [Qigong] explode all trees

**#8**: ("qigong" OR "Qi Gong" OR "Ch'i Kung" OR "baduanjin" OR "yijinjing" OR "liuzijue" OR "wuqinxi"):ti,ab,kw(Word variations have been searched)

**#9**: #7 OR #8

**#10**: MeSH descriptor: [Yoga] explode all trees

**#11**: ("Yoga" OR "hatha yoga" OR "hot yoga" OR "iyengar yoga" OR "pranayama" OR "yoga nidra"):ti,ab,kw(Word variations have been searched)

**#12**: #10or#11

**#13**：(mind-body exercise):ti,ab,kw (Word variations have been searched)

**#14**: MeSH descriptor: [Exercise Movement Techniques] explode all trees

**#15**: (pilates):ti,ab,kw (Word variations have been searched)

**#16**: #14or#15

**#17**:#6or#9or#12or#13or#16

**#18**:#17 and #3

**Chinese database**

**#1:** 太极(tai chi)

**#2:** 太极拳(taijiquan)

**#3:** 气功(qigong)

**#4:** 八段锦(baduanjin)

**#5:** 五禽戏(wuqinxi)

**#6:** 六字诀(liuzijue)

**#7:** 易筋经(yijinjing)

**#8:** 瑜伽(yoga)

**#9:** 普拉提(pilates)

**#10:** 身心运动(mind-body exercise)

**#11:** #1OR#2OR#3OR#4OR#5OR#6OR#7OR#8OR#9OR#10

**#12:** 腰痛(low back pain)

**#13:** 慢性腰痛(chronic low back pain)

**#14:** 非特异性腰痛(nonspecific low back pain)

**#15:** 慢性非特异性腰痛(chronic nonspecific low back pain)

**#16:** #12OR#13OR#14OR#15

**#17:** #11AND#16
