# Supplementary material for: Optimal modes of mind-body exercise for treating chronic non-specific low back pain: Systematic review and network meta-analysis
Source: Front Neurosci. 2022 Nov 17;16:1046518. doi: 10.3389/fnins.2022.1046518 (PMC9713308; doi:10.3389/fnins.2022.1046518)
Supplement: Supplementary file 3 [file Data_Sheet_2.docx]

| **Supplementary materials 3- Definitions of mind-body exercise** | |
| --- | --- |
| Intervention type | Definition |
| Yoga (Kwok et al., 2019) | A mind-body practice that combines physical exercise based on asana (physical poses), breathing, and meditation. It was developed in ancient India.  Benefits of Yoga include both physical and mental improvements. Yoga can improve a wide array of symptoms such as gait, balance, flexibility, strength, pain, anxiety, depression, and sleep. |
| Tai Chi (Song et al., 2017) | A physical exercise delivered through a series of gentle and flowing poses combined with deep breathing and meditation. Tai Chi originated from the ancient Chinese martial art for self-defense and it can improve strength, balance, physical function, cognition, pain, anxiety, and depression |
| Qigong (Song et al., 2017) | An ancient Chinese physical exercise that combines deep breathing and meditation to cultivate the vital energy flow in the body.  Qigong can improve gait, muscle stiffness, balance, coordination, anxiety, depression, and sleep. |
| Pilates (Wells et al., 2012) | Developed by Joseph H. Pilates, Pilates exercise therapy is used to improve an individual’s “flexibility, strength, and body awareness” and it is referred to as a technique that focuses on core stability, posture, breathing, flexibility, strength, and muscle control. |

Kirkwood, G., Rampes, H., Tuffrey, V., Richardson, J. and Pilkington, K. (2005). Yoga for anxiety: a systematic review of the research evidence. *Br J Sports Med,* 39**,** 884-891. doi:10.1136/bjsm.2005.018069

Kwok, J. Y. Y., Kwan, J. C. Y., Auyeung, M., Mok, V. C. T., Lau, C. K. Y., Choi, K. C., et al. (2019). Effects of Mindfulness Yoga vs Stretching and Resistance Training Exercises on Anxiety and Depression for People With Parkinson Disease: A Randomized Clinical Trial. *JAMA Neurol,* 76**,** 755-763. doi:10.1001/jamaneurol.2019.0534

Raub, J. A. (2002). Psychophysiologic effects of Hatha Yoga on musculoskeletal and cardiopulmonary function: a literature review. *J Altern Complement Med,* 8**,** 797-812. doi:10.1089/10755530260511810

Song, R., Grabowska, W., Park, M., Osypiuk, K., Vergara-Diaz, G. P., Bonato, P., et al. (2017). The impact of Tai Chi and Qigong mind-body exercises on motor and non-motor function and quality of life in Parkinson's disease: A systematic review and meta-analysis. *Parkinsonism Relat Disord,* 41**,** 3-13. doi:10.1016/j.parkreldis.2017.05.019

Wells, C., Kolt, G. S. and Bialocerkowski, A. (2012). Defining Pilates exercise: a systematic review. *Complement Ther Med*, 20, 253-262. doi:10.1016/j.ctim.2012.02.005
